# Methamphetamine-Induced Hypoglycemia: A Case Report and Literature Review

**DOI:** 10.7759/cureus.39158

**Published:** 2023-05-17

**Authors:** Henrik Ghantarchyan, Nina Fischer, David Karp, Afshin Hamidi

**Affiliations:** 1 Internal Medicine, Arrowhead Regional Medical Center, Colton, USA; 2 Endocrinology and Diabetes, Arrowhead Regional Medical Center, Colton, USA

**Keywords:** methamphetamine induced hypoglycemia, hirata, insulin, hypoglycemia, methamphetamine

## Abstract

We present a rare case of a 34-year-old male patient with a history of schizophrenia who was found to be persistently hypoglycemic after a positive toxicology screen for methamphetamine. The patient has had multiple admissions to the hospital for persistent hypoglycemia and was then transferred to our in-patient behavioral health unit (BHU). At this time, his toxicology screen was negative for methamphetamines. During his stay in the BHU, he was compliant with his psychiatric medications and was euglycemic despite having a poor appetite until he was discharged home. This patient was shortly readmitted to the hospital and found to be severely hypoglycemic and methamphetamine positive. Here, we present this rare case of methamphetamine-induced hypoglycemia. We emphasize our work-up, treatment, and our suggested theory of why methamphetamines are the likely cause of hypoglycemia.

## Introduction

The well-known psychostimulant methamphetamine is also known to contribute to neurotoxicity [1]. Even though methamphetamine has been extensively studied, the true mechanism of hypoglycemia has not yet been identified. Methamphetamines induce oxidative stress on dopaminergic neurons leading to neuronal inflammation and neuronal degeneration. In regards to other organs, indirect effects on the liver, pancreas, and kidneys have been found. However, currently, there are conflicting studies published on whether methamphetamines result in hypoglycemia vs hyperglycemia [1,2]. Certain counties in the country are more prone to increased methamphetamine use due to lower population socioeconomic status and the stimulant effects of methamphetamine including euphoria, wakefulness, and decreased appetite.

## Case presentation

The patient is a 34-year-old man with schizophrenia, heart failure with an ejection fraction of 20%-25% secondary to non-ischemic dilated cardiomyopathy, and methamphetamine use disorder who presented on numerous occasions with acute encephalopathy and hypoglycemia. He is well known to emergency medical services (EMS) and hospital staff. On arrival, the patient’s blood glucose on the fingerstick was 30 mg/dL. There were no signs of cyanosis in his extremities during the fingerstick or blood draw. The patient’s hypoglycemia was confirmed with a venous blood draw level of 52 mg/dL. To complete the workup for hypoglycemia, the panel of tests was completed, as seen in Table 1, to rule out other differential diagnoses. Liver function tests were obtained, which can be seen in Table 2. A cosyntropin test was also obtained, as seen in Table 3.

**Table 1:** Labs for differential diagnoses for hypoglycemia. pg = picogram, uIU = units of international units, pmol = picomoles, ng = nanogram, u = units, g = gram, dL = deciliter, mEq = milliequivalent, mmol = millimole, mg = milligram, mL = milliliter, ng = nanogram, mIU = milli-international units.

Blood Test Results	Patient Value	Reference Range
Glucagon (pg/mL)	17	11-78
Insulin (uIU/mL)	19.9	≤19.6
Proinsulin (pmol/L)	15.3	≤18.8
C-peptide (ng/mL)	3.04	0.80-3.85
Beta-hydroxybutyrate (mmol/L)	0.09	0.02-0.27
Sulfonylurea	Not detected	
Insulin autoantibody (U/mL)	<0.4	<0.4
Insulin growth factor-1 (ng/dL)	163	53-331
Thyroid-secreting hormone (mIU/L)	1.07	0.35-5.5
Free T4 (ng/dL)	1.11	0.80-1.50
Glucose (mg/dL)	30	65-125

**Table 2:** Liver function tests. U = units, L = liter.

Blood Test Results	Patient Value	Reference Range
Alkaline phosphatase (u/L)	71	35-125
Alanine aminotransferase (ALT) (u/L)	20	5-40
Aspartate aminotransferase (AST) (u/L)	30	5-40
International normalized ratio (INR)	0.99	<1.10
Blood urea nitrogen (mg/dL)	8	8-20
Creatinine (mg/dL)	0.83	0.50-1.50

**Table 3:** Cosyntropin stimulation test. ACTH = adrenocorticotropic hormone, pg = picogram, µg = micrograms.

Blood Test Results	Patient Value	Reference Range
ACTH (at 09:00 am) (pg/mL)	35	6-50
Cortisol at time 0 (µg/dL)	8.30	3-23
Cortisol at 30 minutes (µg/dL)	18.16	3-23
Cortisol at 60 minutes (µg/dL)	23.89	3-23

During each admission, the patient’s urine drug screen (UDS) was positive for only methamphetamine. Our UDS tests for acetone, barbiturate, fentanyl, opiate, phencyclidine, marijuana, and cocaine. The patient was admitted to the intensive care unit (ICU) for hourly monitoring of blood glucose levels as well as 10% dextrose drip administration at a rate of 100 cc/hour for approximately 24 hours. The patient was weaned off the D10 drip and transitioned to a 5% dextrose drip at 50 cc/hour and transferred to the medical floor. Within 24 to 48 hours, the patient began to tolerate the oral diet and the 5% dextrose drip was stopped within approximately 48-72 hours. 

A turning point highlighting the significance of this case was during this patient’s acute hospitalization when the patient was transitioned to inpatient psychiatric care, in which all intravenous medications were stopped. At this point, two separate urine drug screens were negative. During psychiatric care, his appetite was poor. His blood sugars were checked four times a day; he was not hypoglycemic. The patient was discharged and presented to the emergency room (ER) less than 48 hours later with somnolence and hypoglycemia where the UDS on admission was positive only for methamphetamines.

Due to the patient's multiple presentations with hypoglycemia, extensive imaging was done. A computed tomography (CT) of the abdomen and pelvis with and without contrast showed no enhancing lesions with the liver, gallbladder, pancreas, spleen, and adrenal glands unremarkable for any acute or chronic findings. MR abdomen with and without contrast per pancreatic protocol showed no definite pancreatic mass on two separate occasions to rule out insulinoma.

Labs were repeated on multiple visits to confirm the results. An infectious workup was negative including negative blood cultures after five days. Per chart review, the patient’s home medications included sertraline (which was discontinued after two inpatient visits), aripiprazole, carvedilol, and benazepril; however, per the patient’s family member, he had been non-compliant with home medications.

## Discussion

There are many drugs known to induce hypoglycemia including beta-blockers, antiarrhythmics, indomethacin, insulin, and sulfonylureas, among others [3]. The one encountered in our case that is still widely unknown is methamphetamines. On numerous visits for persistent hypoglycemia, the patient was worked up for multiple etiologies possibly precipitating hypoglycemic episodes. During his first admission for hypoglycemia, the patient was on a home dose of sertraline and Abilify for schizophrenia. Sertraline has been noted to induce hypoglycemia via two mechanisms. One way is by impairing the hormonal counter-regulatory response (CRR) of epinephrine and glucagon [4]. The other is by the regulation of liver receptors through 5-hydroxytryptamine activation and inhibition of potassium channels which cause increased insulin sensitivity and production with a concomitant reduction in gluconeogenesis [5]. During one of the patient’s hospitalizations, he was transferred to our behavioral health unit where he was on sertraline and aripiprazole where he did not have any episodes of hypoglycemia.

The patient did not have diabetes. His hemoglobin A1c was 5.5% (reference range: 4.3%-6.1%). Renal and liver diseases were ruled out. Lab values were significant for a high-normal C-peptide, proinsulin, and insulin in the presence of low blood glucose, 52 mg/dL. As the analytical results demonstrate insulin >3 uIU/mL, proinsulin >5 pmol/L, C-peptide >0.6 ng/mL, and beta-hydroxybutyrate <2.7 mmol/L, hypoglycemias are mediated by insulin. The C-peptide and proinsulin were not significantly elevated, which when elevated can increase suspicion of insulinoma and non-insulinoma pancreatogenous hypoglycemia syndrome (NIPHS). Given the concern for insulinoma, the patient underwent a CT scan of the abdomen and pelvis with and without contrast in addition to a magnetic resonance imaging (MRI) with a pancreatic protocol. Both tests did not have any radiologic evidence of insulinoma. Since the hypoglycemia occurred only after methamphetamine use and not after meals, NIPHS was lower on our differential. A 72-hour fast test was not performed.

Another etiology that was considered was insulin autoimmune syndrome (IAS), also known as Hirata disease, which is a rare condition characterized by hypoglycemia due to the presence of increased titers of insulin autoantibodies (IAA) [6]. This was ruled out after insulin autoantibody titers were negative at <0.4 U/mL (normal: <0.4 U/mL). Additional etiologies explored during the hospitalizations included adrenal insufficiency. Adrenal insufficiency was excluded given a normal adrenocorticotropic hormone (ACTH) and a negative cosyntropin stimulation test. Hypothyroidism was also ruled out as one of the possible causes of hypoglycemia. Other etiologies we considered and eliminated included infectious and critical illness, alcohol, and malnourishment despite homelessness. Methamphetamine remains the most likely cause.

The influence of methamphetamines on blood glucose has been studied. One likely explanation is that methamphetamine has a direct effect on the pancreas increasing insulin release. In a study by McMahon et al., plasma insulin in rats increased significantly at 15 minutes and remained elevated significantly above control levels until 60 minutes after methamphetamine injection, causing a rapid decline in glucose levels at the 30-minute mark [7]. In a separate study performed on mice using the analysis of variance (ANOVA) model, it was shown with a P-value of P<0.0001 given a significance level of P<0.05 that methamphetamine does in fact decrease mean blood glucose levels over time [2].

Another possible explanation is the effect that methamphetamine has on glucose transporter protein (GLUT) receptors. In a study looking at human neurons and astrocytes, it was stated that methamphetamine leads to changes in the expression of astrocytic GLUT-1 and GLUT-3 receptors leading to a decreased glucose uptake and a hypoglycemic state in the brain [1]. While there is no data to suggest it has an effect on GLUT-2 receptors in the pancreas, which is required for glucose-stimulated insulin secretion, it could be hypothesized that this could be an alternate process. While the exact mechanism by which methamphetamine lowers blood glucose is vastly unknown, it shines a light on areas requiring further research.

## Conclusions

When working up a patient with persistent hypoglycemia, a thorough workup includes checking insulin levels, C-peptide levels, and obtaining abdominal imaging. We recommend additionally obtaining a urine drug screen to check for amphetamines. We believe that methamphetamine use or abuse can cause significant hypoglycemia, likely from pancreatic-stimulated insulin release.
